# The Range of Bioinclusions and Pseudoinclusions Preserved in a New Turonian (~90 Ma) Amber Occurrence from Southern Australia

**DOI:** 10.1371/journal.pone.0121307

**Published:** 2015-05-13

**Authors:** Annie Quinney, Chris Mays, Jeffrey D. Stilwell, Darla K. Zelenitsky, François Therrien

**Affiliations:** 1 School of Earth, Atmosphere & Environment, Monash University, Clayton, Victoria 3800, Australia; 2 Department of Geoscience, University of Calgary, Calgary, Alberta, T2N 1N4 Canada; 3 Royal Tyrrell Museum of Palaeontology, Drumheller, Alberta, T0J 0Y0 Canada; Institute of Botany, CHINA

## Abstract

A new Turonian amber occurrence, representing the oldest *in situ* amber locality in Australia and the southern-most locality in Gondwana, has recently been discovered in the Otway Basin of Victoria. The amber was collected from petroleum cores and many pieces contain a range of inclusions that can provide information on the depositional history of the resin. To date, one species of fern spore (*Cyathidites minor*) and one species of lycophyte spore (*Kraeuselisporites* sp?) have been conclusively identified in the amber, along with filamentous microorganisms and degraded plant matter. Several samples are also rife with pseudoinclusions as reported recently in other ambers. The abundance of preserved particulate debris and wind dispersed spores suggest that the Otway amber formed subaerially. Furthermore, based on the range of bioinclusions and forms of pseudoinclusions preserved within a single piece of amber, the locus of hardening for individual samples is variably interpreted as occurring in the tree tops, on the tree trunk or on the ground surface. Notably, specific inclusion assemblages are associated with certain colours of amber. By extension, and in accordance with recent studies, amber colour may be indicative of depositional environment. Variation in the environment of solidification may, therefore, be sufficient to account for the broad range of morphological characteristics preserved in a single amber deposit.

## Introduction

The first occurrence of Mesozoic amber from Australia has recently been discovered in Late Cretaceous (Turonian) rocks from offshore and onshore petroleum cores drilled in the Otway Basin, Victoria. Amber is extremely rare in Australia and *in situ* occurrences have only been reported from the Mio–Pliocene Latrobe Valley Coal exposed at Yallourn, Allendale and Lal lal in Victoria [1–3], and in the Eocene lignites exposed at Strahan in Tasmania [4]. Although amber has been discovered on the present day beaches of Cape York [5,6] and along the coast of South Australia to Victoria [2,7], these deposits have been reworked and were likely transported via ocean currents from Cenozoic deposits of Southeast Asia [7,8]. This resin was produced by the Dipterocarpaceae, which first appeared in Southeast Asian tropical rainforests during the Eocene [9] and do not have modern or fossil representatives in Australia [10,11]. Other samples have been described as being sourced from the Cretaceous Wonthaggi coal mine in Victoria [2]; however, a review of the documentation at the Victorian Museum by the authors revealed that the samples were not collected from the coal mine, but from Wonthaggi beach at Cape Paterson. Nuclear magnetic resonance (NMR) analyses of the Wonthaggi samples revealed a strong similarity to resins produced by the Dipterocarpaceae [2,12]. Therefore, we suggest that these anomalous samples were also transported via ocean currents from Cenozoic deposits of Southeast Asia. Other occurrences of Gondwanan amber are limited, but are known from Brazil [13,14], Argentina [14], South Africa [15], Ethiopia [16], and New Zealand [2]; however, these samples were deposited at low to middle latitudes. Ergo, not only is the Otway amber the oldest *in situ* amber found in Australia, but it is also the first high latitude amber from Gondwana, for which few amber deposits are known.

Relative to the Southern Hemisphere, Northern Hemisphere amber deposits are common and several Turonian localities have been discovered in Siberia, Russia, the United States and France [14]. Some of these localities are important paleontological sites, preserving numerous examples of arthropod and plant remains, e.g. [17,18]. In addition to macroscopic bioinclusions, other Cretaceous ambers also contain abundant microorganisms such as amoebae, e.g. [19,20–22]; ciliates, e.g. [23]; cyanobacteria, e.g. [24,25]; algae, e.g. [20,22]; filamentous bacteria, e.g. [16,26,27]; and fungi, e.g. [16,20,26,27,28]. Although many of these identifications are unambiguous and based on preserved diagnostic structures, others are based solely on the gross morphology of the inclusion [29]. Recently, it has been suggested that some of the purported microorganisms in amber are not bioinclusions, but are simply artefacts formed during exudation of the resin [19,21,30].

Because Mesozoic fossil localities are rare in Australia, the Otway amber has the potential to provide new data on the diversity of life that existed in the high latitude Turonian austral forest. Here, we describe the morphological characteristics and range of inclusions preserved in the oldest amber occurrence from Australia and the southern-most amber occurrence from Gondwana. In turn, inclusion assemblages are used to infer the depositional histories of the amber samples. Given the recent reinterpretation of numerous amber inclusions as artefacts or pseudoinclusions [21,30,31], careful evaluation of the inclusions in the Otway amber is warranted.

## Geologic Background

**Fig 1:**
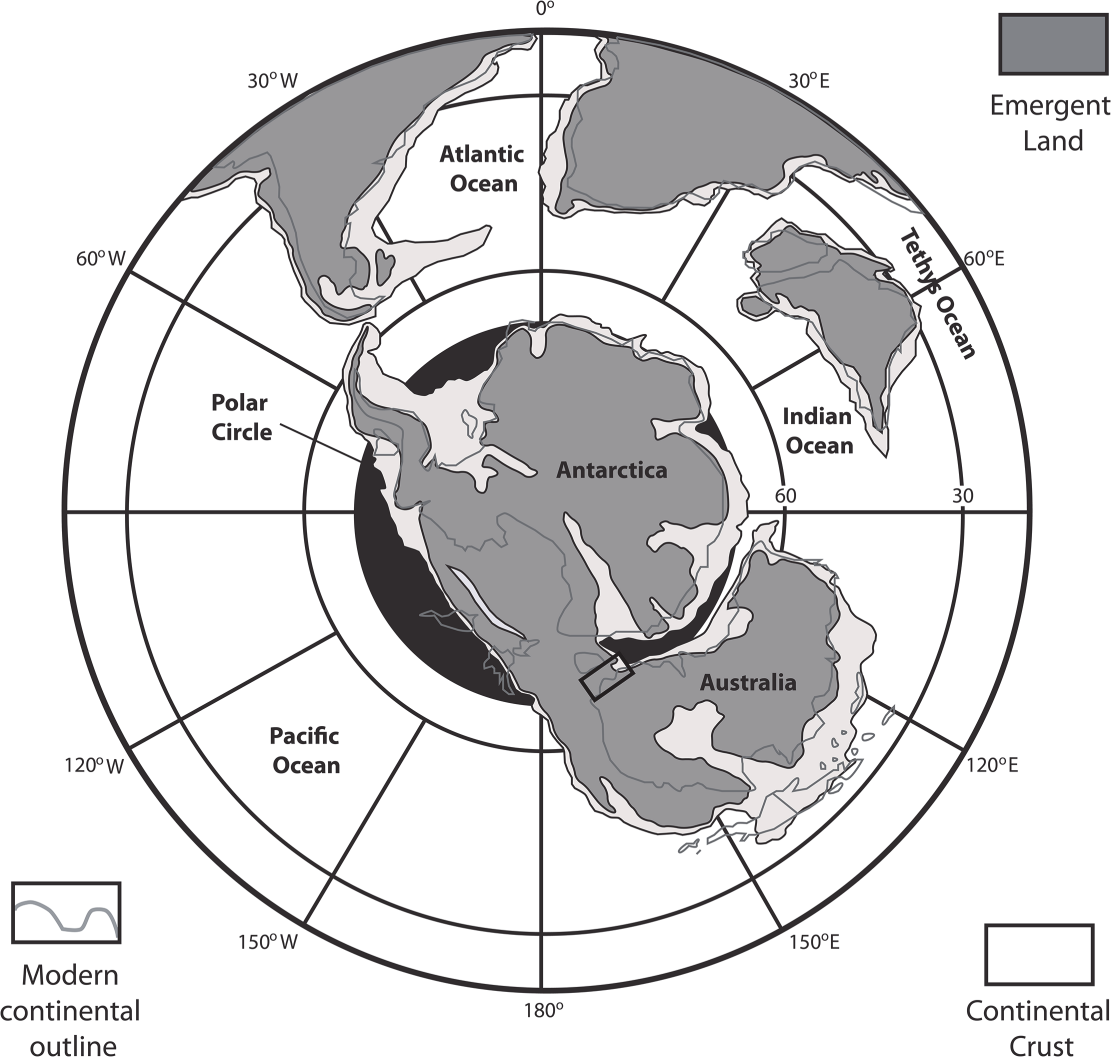
Turonian palaeogeographic reconstruction of the continents from a South Polar perspective. Black box over Australia indicates study area. Modified from [35].

The Otway Basin began as a Mesozoic rift basin that developed during the separation of Australia from Antarctica. Although Australia maintained a connection to Antarctica throughout the Turonian, rifting progressed from west to east as Australia moved northward [32]. During that time, the southern margin of Victoria hovered around the boundary of the Antarctic Circle between palaeolatitudes of approximately 60–70°S (see Fig 1) [33,34]. Continued rifting led to the complete separation of Australia from Antarctica by the Eocene and culminated in the final breakup of Gondwana [34].

The amber was deposited in the Turonian Flaxman and Waarre formations of the Upper Cretaceous Sherbrook Group [36–41]. The Sherbrook Group consists of a clastic wedge of sediment that thickens rapidly to the south [42], filling the rift basin. The creation of rift basins led to the development of marginal marine to fully marine depositional environments along the present-day coast of Victoria. As a result, a series of deltas developed along the Turonian coastline [43]. The distribution of wells containing amber is concentrated on, or adjacent to, these ancient deltas. Equivalent formations do not crop-out onshore in Victoria as extensive erosion has removed a substantial portion of the Upper Cretaceous succession [44]. As such, amber exploration for this time period is restricted to drill cores.

## Sample Collection and Preparation

**Fig 2:**
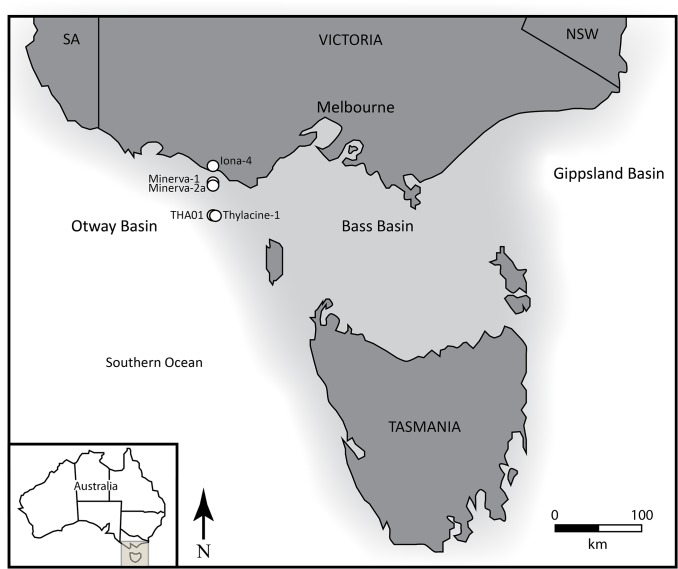
Study area map within the Otway Basin (grey box) off the coast of Victoria, Australia (inset). White dots indicate amber-bearing well locations including well names. Dark grey shading represents modern land; light grey represents continental crust.

Seventy rock samples containing one or more pieces of amber (n = 267) were collected from six petroleum cores drilled off the coast of Victoria, Australia in the Otway Basin (Fig 2). Amber was collected from the cores either manually using forceps or by cutting a small piece from the core such that the amber remained embedded in matrix. Excess matrix was removed using a rock saw, and the amber was cleaned and polished using wet silicon carbide abrasive paper at successively finer grit (FEPA P 600–3000 or 25.8–7 μm) to expose a viewing surface. Like many Cretaceous ambers, e.g. [45,46], the Otway amber is brittle and many samples were either collected as broken fragments from the core or burst during extraction. In order to preserve large pieces intact and aid in visual inspection, select samples were cleaned and embedded in Epotec epoxy resin following the method of [47]. Each whole piece and fragment of burst amber was examined using transmitted light under a Leica M80 and a Leica DM2500P microscope fitted with a Leica DFC 290 HD camera and photographed using the Leica Application Suite (LAS) software, version 3.8 and the Montage Multifocus module. In cases where multiple amber pieces were contained within a single rock sample, some of the amber was left embedded in matrix for preservation. Permission to collect samples from core was granted by the Department of State Development, Business and Innovation (State Government, Victoria), the Department of Infrastructure, Energy and Resources (Tasmanian Government), and by Weatherford Laboratories (Brisbane, Australia). Samples are curated at the Melbourne Museum, which is part of Museum Victoria (institutional abbreviation: MV) and accession numbers are outlined in Table 1.

**Table 1:** Sample accession numbers.

Sample	Accession Number	Sample	Accession Number
THA01 2412.7	P251446	THA01 2531.11	P251470
THA01 2451.7	P251447	THA01 2531.47	P251471
THA01 2466.89-2D2-1	P251448	THA01 2532.9	P251472
THA01 2466.89-2D2-2	P251449	THA01 2554.86	P251473
THA01 2466.89–005	P251450	Minvera-1 1829.6	P251474
THA01 2466.89–009	P251451	Minvera-1 1836.9	P251475
THA01 2466.89	P251452	Minvera-1 1837.5	P251476
THA01 2466.9	P251453	Minvera-1 1838.45	P251477
THA01 2467.8	P251454	Minvera-2a 1840.0	P251478
THA01 2468.1	P251455	Minvera-2a 1843.2	P251479
THA01 2474.4	P251456	Minvera-2a 1857.43	P251480
THA01 2500.25	P251457	Minvera-2a 1860.4	P251481
THA01 2500.65	P251458	Minvera-2a 1860.5	P251482
THA01 2500.7	P251459	Minvera-2a 1867.7	P251483
THA01 2501.4	P251460	Minvera-2a 1880.8	P251484
THA01 2516.25	P251461	Minvera-2a 1941.6	P251485
THA01 2520.34	P251462	Minerva-2a 1945.2	P251486
THA01 2529.33	P251463	Minerva-2a 1956.7	P251487
THA01 2529.43	P251464	Iona-4 1448.8	P251488
THA01 2529.65	P251465	Iona-4 1456.43	P251489
THA01 2530.9	P251466	Iona-4 1459.3	P251490
THA01 2531.06	P251467	Iona-4 1464.7	P251491
THA01 2531.07	P251468	Iona-4 1465.6	P251492
THA01 2531.07–007	P251469		

Sample numbers (given as well location, depth of collection (m)-sample number) and associated accession numbers. All samples are housed at the Melbourne Museum, part of Museum Victoria (MV).

## Macroscopic Features of the Amber

**Fig 3:**
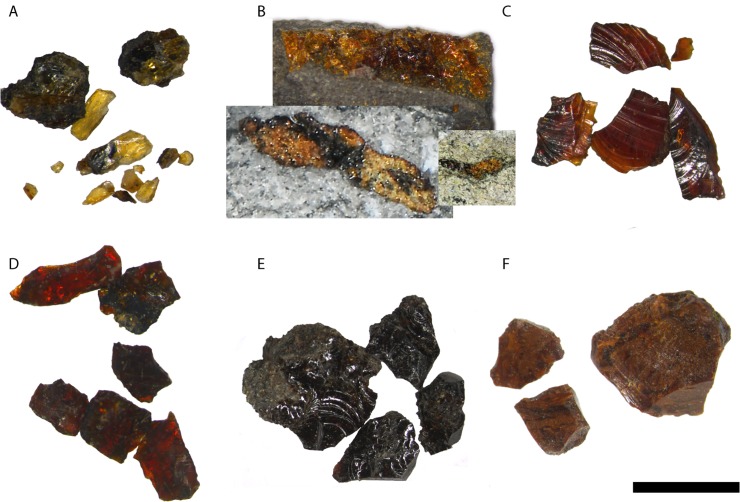
Colours of Australian amber. A) burst piece of yellow amber, some still embedded in matrix, sample THA01 2529.43; B) Top: Partial nodule of orange amber embedded in matrix, sample THA01 2451.7; Bottom left: cross-section through orange amber nodule containing organic matter, sample Minerva 2a 1857.43; Bottom right: cross-section through orange amber droplet, (not collected) Minerva 2a 1923.5; C) fragments from burst piece of dark orange amber sample Minerva-1 1837.5; D) fragments from burst piece of red amber, sample Minerva 2a 1843.2; E) fragments from burst piece of dark brown amber sample Minerva 2a- 1945.2; F) fragments from burst piece of milky brown amber, sample Minerva 2a 1956.7. Scale bar: 4 mm.

The Otway amber is variably coloured from pale yellow to dark red, yet most pieces exhibit exceptional transparency (Fig 3). Rare samples, however, were dark brown and opaque. The amber ranged in size from sub-millimetre flecks to pieces up to three centimetres in length. Two forms of amber are common in the Otway Basin: abundant, small (0.5–5 mm) droplets and uncommon, larger (1–3 cm) nodules. The droplets are often preserved in carbonaceous mudstones and along bedding planes of thin coal laminations. The droplets are typically lens-shaped, with the short axes oriented perpendicular to bedding planes. By contrast, the nodules tend to occur in sandy siltstones and sandstones, occasionally within lenses of dispersed organic matter. Rarely, large (2–3 cm) discoid amber pieces with irregular margins were observed in carbonaceous mudstones. These pieces were roughly planar with the short axes oriented perpendicular to bedding. The droplets are inferred to represent the morphology of the original resin flows, e.g. [26], that were subsequently compressed during burial. Similarly, the larger discoid pieces likely represent more substantial flows that underwent compaction. By contrast, the nodules likely underwent reshaping and fragmentation during transport, e.g. [26]. Because these pieces tend to be more equant, and because sandstones are less compressible than mudstones, the effects of compaction are considered minor.

## Description of Inclusions

Abundant inclusions with diverse forms were observed in many samples of Turonian Otway amber when viewed under the microscope. These inclusions are grouped into seven categories: 1) plant spores; 2) filamentous microorganisms; 3) degraded plant matter; 4) spherical inclusions; 5) ovoid and fusiform inclusions 6) irregular filamentous inclusions; and 7) amorphous inclusions.

### Plant spores

**Fig 4:**
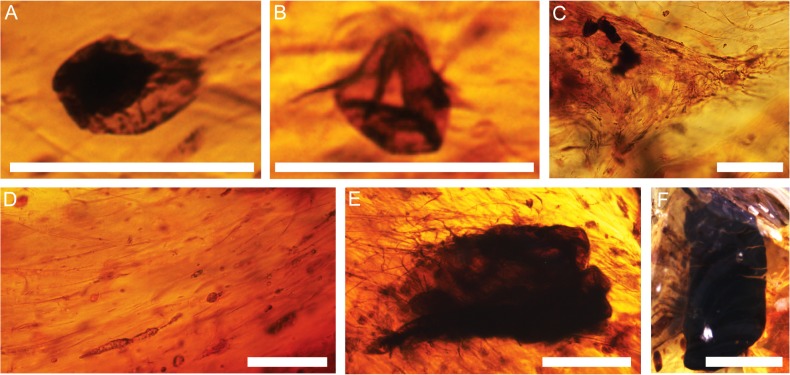
Examples of bioinclusions from Otway amber. (A) Probable specimen of *Kraeuselisporites* Raine, 2008, Sample THA01 2466.89 2D2-1. (B) *Cyathidites minor* Couper, 1953, Sample THA01 2466.89 2D2-1. (C) Randomly oriented network of filamentous microorganisms, Sample THA01 2466.89 2D2-2. (D) Mutually aligned filamentous microorganisms pictured with ovoid and fusiform inclusions, Sample THA01 2466.89 2D2-1. (E) Degraded organic matter exhibiting cracks along outer margin, Sample THA01 2466.89 009–1. (F) Degraded organic matter with attached filamentous microorganisms, Sample THA01 2531.07–007. Scale Bars: 100 μm (A–D); 50 μm (E); 200 μm (F).

Two plant spores were identified in the Otway amber. Both spores occur within the same piece of amber, along with mutually aligned filamentous microorganisms, decayed organic matter, irregular filamentous and amorphous inclusions. The first specimen consists of a large (equatorial diameter: 77.5 μm including zona, 47.5 excluding zona), trilete, acavate, zonate microspore with a convexly sub-triangular amb (excluding zona) or an ovate amb (including zona; Fig 4A). The thick nexine (1.5–2.5 μm) has indistinct (scabrate/ granulate?) sculpture. The zonate sexine is thin (0.5–1.0 μm) and readily folded.

The second specimen consists of a small trilete microspore (equatorial diameter 39.5 μm, 42.5 μm; polar diameter 21 μm) with a sub-triangular amb, straight to concave sides and rounded apices (Fig 4B). The proximal surface has stronger convexity than the dorsal side. Simple, unthickened leasurae are between half and three quarters of the spore radius in length. The exine is unsculptured, single-layered and approximately 1.5 μm thick.

### Filamentous microorganisms

Filamentous microorganisms are completely entombed in amber and consist either of randomly oriented, ramified networks or mutually aligned filaments (Fig 4C & 4D). Randomly oriented filamentous microorganisms may co-occur with spherical inclusions and decayed organic matter, whereas mutually aligned filaments often co-occur and are aligned with other elongate inclusions (i.e. fragments of decayed organic matter or any of the ovoid, fusiform, irregular filamentous and/or amorphous inclusions discussed below). The filaments may be straight, irregular and/or spiralled and appear continuous. Individual filaments range in diameter from approximately 1–5 μm and possess either a consistent diameter along the branch or exhibit occasional swellings. The colour of the filaments varies with the colour of the amber, appearing slightly darker than the embedding medium.

### Degraded plant matter

Degraded plant matter preserved in the amber varies substantially in form, lacking a characteristic or common shape. The inclusions are often completely entombed in amber, but occasionally project on to the surface. The inclusions may co-occur with any of the other inclusions but are most often associated with filamentous microorganisms, which originate on the organic matter and extend into the surrounding amber (Fig 4E). The semi-opaque or opaque inclusions may have smooth, well-defined edges or irregular margins. Often, the outer surfaces are disseminated with thin cracks that originate from the margin of the inclusion (Fig 4F). The opacity of these inclusions inhibits examination of potential internal structures, and the degree of degradation has destroyed potential surficial structures. The size of the inclusions ranges from 150–1000 μm, measured for the longest dimension. Colour varies from orange-brown to black, regardless of the background colour of the amber.

### Spherical inclusions

**Fig 5:**
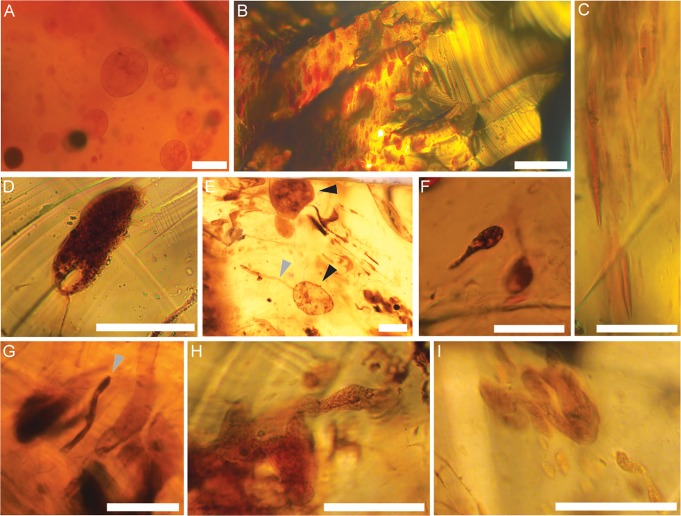
Examples of other inclusions from Otway amber. (A) Spherical inclusions (Type A pseudoinclusions) with internal vesiculation, Sample Minerva-1 1836.9. (B) Concentration of ovoid inclusions (Type B pseudoinclusions) on the left side (dark orange) and no inclusions of the right side (lighter orange), Sample THA01 2451.4. (C) Mutually aligned fusiform inclusions (Type B pseudoinclusions), Sample THA01 2451.7. (D) Ovoid inclusion (Type B pseudoinclusion) with single vesicle; Sample THA01 2466.89 2D2-2. (E) Ovoid inclusions (black arrows; Type B pseudoinclusions); lower ovoid inclusion (Type B pseudoinclusions) is stacked on top of an irregular filamentous inclusion (grey arrow; Type D pseudoinclusion); pictured with filamentous microorganisms, Sample THA01 2466.89 2D2-2. (F) Vesicular ovoid inclusion (Type B pseudoinclusion) with projection, Sample THA01 2466.89 2D2-1. (G) Irregular filamentous inclusion (grey arrow; Type D pseudoinclusion) surrounded by mutually aligned filamentous microorganisms and ovoid inclusions (Type B pseudoinclusions), Sample THA01 2466.89 005. (H) Amorphous inclusion (Type E pseudoinclusion), Sample THA01 2466.89 2D2-2. (I) Amorphous inclusions (Type E pseudoinclusion) surrounded by mutually aligned ovoid inclusions, Sample THA01 2466.89 2D2-2. Scale bars: 50 μm (A, F); 200 μm (B); 100 μm (C–E, G–I).

Smooth, rounded spherical inclusions are completely entombed in amber. The number of spherical inclusions preserved within a single sample ranges from a few to several hundred. Owing to their shape, spherical inclusions display no preferred orientation. The inclusions commonly co-occur with decayed organic matter and occasionally with randomly oriented filamentous microorganisms, but never with fusiform, ovoid, irregular filamentous or amorphous inclusions. The margins of the inclusions are smooth and well-defined, and the interiors may be either vesiculated or structureless (Fig 5A). The inclusions exhibit a range of sizes, measuring between 5–200 μm in diameter. Colour of the inclusions varies from pale yellow-orange to dark orange-brown, regardless of the background colour of the amber.

### Fusiform and ovoid inclusions

Fusiform and ovoid inclusions are elongate and, in the case of the former, have tapered ends. These inclusions are completely embedded in resin and occur in clusters of a few to several hundred mutually aligned inclusions. Often, the inclusions co-occur, and are aligned with, filamentous microorganisms, elongate fragments of decayed organic matter, irregular filamentous inclusions and amorphous inclusions. Fusiform and ovoid inclusions may have smooth exteriors (Fig 5B–5E) or may have horns or projections that extend out from the surface (Fig 5F). Like spherical inclusions, some fusiform and ovoid inclusions have vesiculated interiors, whereas others appear structureless. The inclusions range from approximately 5–200 μm in maximum diameter. Colour varies from pale yellow-orange to dark orange-brown, regardless of the background colour of the amber.

### Irregular filamentous inclusions

Irregular filamentous inclusions are elongate, unramified inclusions that are completely embedded in resin (Fig 5E & 5G). These inclusions commonly co-occur, and are aligned with, other elongate inclusions including filamentous microorganisms, elongate fragments of decayed organic matter, and ovoid, fusiform and amorphous inclusions. Unlike other inclusions, irregular filamentous inclusions tend to be isolated and do not form large clusters. The margins of these inclusions are well defined and the interiors tend to be structureless. Inclusions range from 5–25 μm in width by 90–225 μm in length. Colour varies from pale yellow-orange to dark orange-brown, regardless of the background colour of the amber.

### Amorphous inclusions

Amorphous inclusions lack a distinct or characteristic form, occur singly and, as above, are typically aligned with other elongate inclusions preserved within the sample. The margins of the inclusions tend to be irregular, and the interiors are often speckled and/or finely vesiculated (see Fig 5H & 5I). Considerable variations in size are exhibited by the amorphous inclusions, ranging from approximately 10–200 μm in length measured for the longest dimension. The inclusions exhibit a range of colours from pale orange to dark brown, regardless of the background colour of the amber.

## Interpretation of Inclusion Types

Inclusions in amber from the Otway Basin consist predominantly of pseudoinclusions, although some bioinclusions are also preserved. Two spores were conclusively identified in the Otway amber, although low level taxonomic identifications of microorganisms and plant matter could not be made for the remaining bioinclusions due to a general absence of diagnostic features. Many inclusions show irregularities in form and inconsistencies in size that, when considered in conjunction with the lack of diagnostic characteristics, indicate a non-biological in origin. These are interpreted as structural artefacts created during exudation of the original resin, e.g. [19,21,30,31]. Although many of the artefacts bear a strong semblance to protists (termed pseudo-protists; [30]), several other forms were also identified in the Otway amber. Here, all forms of artefacts in the Otway amber are collectively referred to as pseudoinclusions.

### Bioinclusions

#### Plant spores

The first specimen is tentatively interpreted as *Kraeuselisporites* sp. (Fig 4A and Table 2). As noted by Raine [48], *Kraeuselisporites-*like zonate spores with distal sculpture are comparable to extant members of the lycophyte group Selaginellaceae [49]. According to Scheuring [50], *Kraeuselisporites* can be distinguished from similar zonate genera (such as *Perotrilites* Erdtman, 1947 emend Evans, 1970 ex Couper, 1953) [51, 52] by the presence of a distally sculptured sexine that consists of isolated elements or an infrareticulate structure. Features of this specimen, such as the size and loose-fitting zona, are comparable to some specimens of *Perotrilites granulatus* Couper, 1953, as recorded from various mid-Cretaceous (Albian-Cenomanian) strata of New Zealand, e.g. [53,54]. These features are also common to *Kraeuselisporites alexii* Raine, 2008, which have been recorded from Turonian to Campanian strata of New Zealand. The sexine to nexine diameter ratio, however, is more in line with the *Kraeuselisporites*. The crucial details of the sculpture and laesurae are not presently discernible on the specimen described herein; as such, a definitive designation of this specimen to *K*. *alexii* cannot be made. Although lycophyte spores have been previously recorded as inclusions in Cenomanian amber of eastern Africa [16], they are under-represented in reports of fossil resin.

**Table 2:** Types and interpretations of inclusions found in the Otway amber.

Inclusion type	Interpretation	Amber Colour	Relative Abundance	Sample ID [Well name: depth (m)]
Spores	*Kraeuselisporites* sp., *Cyathidites minor*	Orange	Rare	THA01: 2466.89
Filamentous Microorganisms	Fungal hyphae/ Filamentous bacteria	Orange, Red?	Uncommon	THA01: 2466.89, 2467.8, 2520.34, 2531.07
Decayed Organic Matter	Type C*	Yellow, Orange, Dark- orange, Red, Brown	Common	THA01: 2412.7, 2451.7, 2466.89, 2466.9, 2474.4, 2500.65, 2500.7, 2501.4, 2516.25, 2529.33, 2531.06, 2531.07, 2531.11, 2531.47; Minerva-1: 1837.5, 1838.45; Minerva-2a: 1843.2, 1857.43 1860.5, 1941.6; Iona-4: 1448.8, 1456.5, 1459.3 1464.4, 1464.7, 1465.6
Spherical	Type A*	Yellow, Orange, Dark- orange, Red	Abundant	THA01: 2451.7, 2500.25, 2500.65, 2501.4, 2529.33, 2529.65, 2530.9, 2531.06, 2531.11 Minerva-1: 1829.6, 1836.9, 1838.45 Minerva-2a: 1840.0, 1843.2 1857.43, 1860.4, 1860.5, 1867.7 1880.8; Iona-4: 1459.3, 1465.6
Ovoid	Type B*	Orange, Dark- orange	Common	THA01: 2412.7, 2451.7, , 2466.89, 2466.9, 2468.1, 2529.43, 2531.07, 2532.9, 2554.86 Minerva-1: 1838.45; Minerva-2a: 1860.5, 1941.6; Iona-4: 1464.7
Filamentous Irregular	Type D	Orange	Rare	THA01: 2466.89, 2531.07; Minerva-1: 1838.45
Amorphous	Type E	Orange	Rare	THA01: 2451.7, 2466.89

Upper bold box indicates bioinclusions; lower bold box indicates pseudoinclusions. Relative abundances are based visual assessments of inclusion types in the entire sample set. For a breakdown of relative abundances per amber colour, see Table 3.

*Interpretations based on the pseudo-protist classification scheme of Girard et al. [30].

The second specimen is indistinguishable from *Cyathidites minor* (Fig 4B and Table 2), a spore commonly attributed to the fern groups Cyatheaceae or Dicksoniaceae [55,56,57]. These spores are pervasive in Cretaceous sediments of Eastern Gondwana, including the Antarctic Peninsula [58], New Zealand [55], southeast Australia [59] and Western Australia [60,61]. Although this taxon is the most commonly identified fossil spore in Mesozoic sediments of Gondwana, this is the first known record of *C*. *minor* preserved within Mesozoic amber. This result is somewhat unexpected, given that fern spores are prolific, wind dispersed and regularly found as inclusions in amber, e.g. [16,62,63]. Although no comparable spores of this group have been found in Mesozoic Gondwanan amber, cyatheaceous stellate hairs have been identified in Cenomanian amber from Ethiopia [16]. Because of the broad biostratigraphic range of *C*. *minor*, this species does not provide any further constraints on the age of the amber.

#### Filamentous microorganisms

The filamentous microorganisms present in the Otway amber are identified as either filamentous bacteria (Actinomycetes) or fungal hyphae (Table 2). The forms of modern and fossil fungal hyphae and filamentous bacteria are diverse and overlap, making it difficult to distinguish these organisms based solely on general morphology (see [26] for a review). In the absence of diagnostic reproductive structures and in light of the considerable overlap in the morphological characteristics of fungal hyphae and filamentous bacteria, low level taxonomic identifications of the filamentous microorganisms preserved in the Otway amber are not made here.

All of the Australian filamentous microorganisms are completely entombed in amber (Fig 4C–4E). Aligned filaments (Fig 4D) are common and likely represent inclusions entrained in liquid resin and oriented in the direction of flow, e.g. [64]. Randomly oriented ramified networks that extend into the resin mass (Fig 4A) likely represent *in situ* colonization by organisms and consumption of immobile liquid resin, e.g. [65,66, 67]. Examples where the organisms originate on decayed organic matter and extend out into the amber (Fig 4E) likely represent encapsulation of infected plant tissue, e.g. [66], and secondary accumulation in the liquid resin, e.g. [68]. Although filamentous bacteria and/or fungal hyphae can also colonize the surfaces of resin and amber before, during or after burial [65], no examples of this were found in the Otway amber.

#### Degraded plant matter

Dark-coloured, opaque inclusions exhibiting a range of forms and sizes are interpreted as degraded organic matter (e.g., [30]). Although Girard et al. [30] classified similar inclusions as Type C pseudo-protists, we suggest that the organic origins of the inclusions permits classification under bioinclusions (Table 2). Cracks in the inclusions (Fig 4F) are interpreted as desiccation cracks that formed during decay prior to entrapment and potentially enhanced by dehydration during fossilization, e.g. [69–73]. As mentioned above, the fact that filamentous inclusions originate on the organic matter and extend into the resin mass (Fig 4E) suggests that the organic matter was infected prior to encapsulation, e.g. [66].

### Pseudoinclusions

#### Spherical, ovoid and fusiform inclusions

The external morphology of the spherical, fusiform and ovoid inclusions, in conjunction with internal vesiculation, superficially resembles the gross morphology of many testate protists, e.g. [74–76], although here they are interpreted as pseudoinclusions. The preservation of delicate, vacuole-like structures (i.e. vesiculated interiors) in the absence of readily preserved, robust organelles such as nuclei is odd, e.g. [30,77]. Furthermore, hundreds to thousands of these inclusions, exhibiting a wide range of sizes, are preserved within a single piece of Otway amber. As indicated by Girard et al. [30], this type of assemblage is not representative of true protist ecology, suggesting that these inclusions are structural artefacts corresponding to the Type A (spherical; Fig 5A) and Type B (fusiform and ovoid; Fig 5B & 5C) pseudo-protists of Girard et al. [30] (Table 2).

Ovoid inclusions with horns or projections extending from the surface of the inclusion (Fig 5E & 5F) superficially resemble germinating fungal spores and/or testate amoebae with cell plasma extending out from a broken shell. Reports of similar inclusions in other ambers can be found in the literature, e.g. [21,78,79]. The Australian inclusions, however, tend to be large (50–200 μm) and fall outside the average size range (2–20 μm) of modern fungal spores of [80]. Although the inclusions do fall within the size range (10–300 μm) of testate amoebae [81], without the apparent preservation of apertures or pores, one cannot conclusively make a taxonomic identification. Further, the strong similarity of the main “body” of these inclusions to other ovoid and fusiform pseudo-protists suggests that they, too, represent protist-like pseudoinclusions. The projections also align with flow, indicating that, like the ovoid pseudoinclusions to which they are attached or associated, the projections were also deformed during movement of the liquid resin. Notably, in the case of Fig 5E, the ovoid inclusion and the projection actually represent two distinct, overlapping inclusions (an ovoid inclusion and an irregular filamentous inclusion), which can be discerned in stepwise photomicrographs.

#### Irregular filamentous inclusions

Some filamentous inclusions in the Otway amber exhibit irregularities in shape and size (Fig 5E & 5G). These inclusions are unramified and tend to have large diameters (up to 20 μm), which exceed the average diameters of fungal hyphae and filamentous bacteria, e.g. [82]. The inclusions are always aligned with other elongate inclusions, suggesting orientation with flow of the liquid resin. Similar inclusions described by Ragazzi and Schmidt [31] have been identified as pseudoinclusions based on irregularities in form and size and a general absence of diagnostic features. As a result, the irregular filamentous inclusions in the Otway amber are also interpreted as pseudoinclusions (herein referred to as Type D; Table 2), elongated and aligned by flowing resin.

#### Amorphous inclusions

Amorphous inclusions (Fig 5H & 5I) lack consistencies in form and size and grossly resemble naked amoebae; however, they do not contain any diagnostic features that could be used for taxonomic identification, see [19]. Because amorphous inclusions within a single piece of amber all likely formed at the same time, see [30], and are aligned with other elongate inclusions within the sample, amorphous inclusions (herein referred to as Type E pseudoinclusions; Table 2) are inferred to represent the amalgamation of multiple Type B and D pseudoinclusions in flowing resins.

#### Distribution of inclusions based on amber colour

Orange amber is the most common colour present, representing approximately 65% (n = 173) of the samples. All five types of pseudoinclusions occur in orange amber, but Type B are the most abundant (Table 3). The two plant spores, *Cyathidites minor* and *Kraeuselisporites* sp?, were also found in the orange amber, along with most (if not all) of the documented filamentous microorganisms.

**Table 3:** Determining the history of resin exudation and solidification based on the relationship between the relative abundances of pseudoinclusion types and amber colour.

This study						Girard et al., 2011				
	Yellow	Orange	Dark Orange	Red	Brown	Yellow	Honey	Milky	Red	Litter
Type A	+	++	+++	++	n.o.	+	+++	+	+++	+
Type B	-	+++	+	-	n.o.	-	+	+++	+	-
Type C	+	++	++	++	+++	+	++	+	++	+++
Type D	-	+	+	-	n.o.	n.d.	n.d.	n.d.	n.d.	n.d.
Type E	-	+	+	-	n.o.	n.d.	n.d.	n.d.	n.d.	n.d.
Mobility	I	I,M*	I*,M	I	M	I	I*,M	M	I*,M	I
Environment	B	B,T*,G	B,T*	B,T*	G	B	T	R	P	G

–: not present; +: uncommon; ++: common; +++: abundant, n.o.: not observed (due to opacity), n.d.: not described. Mobility (of resin); inferred from the pseudoinclusion assemblage preserved in each colour of amber (I: immobile; M: mobile). Environment (of solidification); inferred from inclusion assemblage and/or amber morphology. (B: upper tree branches; T; tree trunk; G: ground surface; R: underground (root resin); P: pond). Inclusions were not observed in milky brown amber (sample ID: Minerva-2a 1956.7); therefore, it is not included in the table. Modified from [30].

*Most common interpretation based on pseudoinclusion abundances

Yellow amber is the second most common type of amber collected, representing approximately 15% (n = 41) of the samples. As in Girard et al. [30], inclusions are rare in the yellow amber and restricted to Type A and C pseudoinclusions (Table 3).

Dark orange amber represents 8% of the amber collected (n = 22). Type A pseudoinclusions are particularly abundant in the dark orange amber, but Types B–E are also present (Table 3). Colour often varies within a single piece of amber and is associated with differences in the abundance and/ or types of pseudoinclusions (Fig 5B). In these cases, abundant Type A or B pseudoinclusions were observed in the dark orange areas and few to none were observed in the lighter orange areas. Exceptionally, the occurrence of a dark orange rim around a lighter orange core is inferred to represent weathering and oxidation of the outermost surface, e.g. [83], as no difference in the abundance or types of inclusions exists between the core and the rim.

Red amber represents approximately 10% (n = 26) of the samples collected. Inclusions are not uncommon in the red amber and are largely restricted to Type A and C pseudoinclusions (Table 3), although one possible example of filamentous microorganisms may exist.

The least common type of amber is brown amber, representing less than 2% (n = 5) of the samples collected. Because of the largely opaque nature of brown amber, inclusions are difficult to observe. Opacity is related to an abundance of organic matter (Type C pseudoinclusions) and particulate debris in most samples (Table 3), which can be observed in thin amber fragments. One of the opaque amber samples (well name: Minerva-2a; depth 1956.7m) has a milky brown appearance; unfortunately, the high degree of opacity of this sample precludes investigation for inclusions using light microscopy.

#### Implications of inclusions and colour for amber depositional history

The range of colours exhibited by amber from a single deposit have been variably interpreted and may represent: (a) differences in depositional and post-depositional weathering or oxidation [83,84], or (b) difference in the types and abundances of occluded materials [84,85]. Additionally, Girard et al. [30] noted that certain types of pseudo-protists appear to be associated with specific colours of Charentes amber and used this information to infer depositional environments. Although the results presented here largely agree with the findings of Girard et al. [30], some notable differences exist.

Like Charentes amber, yellow amber from the Otway Basin is devoid of bioinclusions and particulate debris, and only preserves a limited number of Type A pseudoinclusions (Table 3). In accordance with the interpretation of Girard et al. [30], yellow amber from the Otway Basin is inferred to represent resin exuded by tree branches or trunks and hardened in place. Likely, this resin was exuded on the upper portions of trees, where less particulate debris can be wind-transported, e.g. [86].

Red amber from the Otway Basin also displays a similar distribution of pseudoinclusions to Charentes amber (Table 3), consisting predominantly of a variable number (from none to several hundred) of Type A pseudoinclusions. Unlike Charentes amber, however, no Type B pseudoinclusions were observed. Girard et al. [24,30] commonly found Type A pseudoinclusions in red amber with a cortex comprised of abundant freshwater filamentous microorganisms. As a result, Girard et al. [30] suggested that spherical pseudoinclusions were common in resins that were exuded into ponds and dried slowly. In the case of the Otway amber, however, no aquatic microorganisms were found in association with Type A pseudoinclusions, despite the deltaic depositional environment. Furthermore, both Girard et al. [30] and the present study documented spherical pseudoinclusions in yellow amber, suggesting that Type A pseudoinclusions can also form subaerially. All amber possessing Type A pseudoinclusions are, therefore, simply inferred to represent immobile resins that hardened at the site of exudation–conditions that could be achieved subaerially (as in the case of the yellow and red Otway and yellow Charentes amber) or subaqueously (as in the case of the red Charentes amber).

Orange and dark orange ambers exhibit major variation in both the number (from none to several hundred) and types (A–E) of pseudoinclusions preserved, which may or may not be associated with bioinclusions. Girard et al. [30] did not describe orange or dark orange amber, however, the distribution of pseudoinclusions in the dark orange amber closely resembles that of “honey” amber (Table 3). Although all five types of pseudoinclusions were documented from different samples of orange and dark orange amber, the abundance of Type A pseudoinclusions in the dark orange amber suggests that, like yellow and red amber, dark orange amber largely solidified in place. By contrast, the predominance of Type B pseudoinclusions in orange amber suggests at least some degree of transport away from the locus of exudation, e.g. [30]. Additionally, many samples of orange, dark orange and red amber contain abundant particulate debris. These samples are inferred to have formed lower down on the tree trunk, e.g. [30], where wind transported dust and debris are more likely to be trapped [86].

Brown amber (and the occasional sample of orange amber) is filled with degraded organic matter that often extends from the core to the surface of the sample. The abundance of poorly preserved organic matter in these samples suggests that the resin dripped on to, and engulfed, organic matter from the topsoil (i.e. litter amber; [30,87]). Marine microorganisms have been observed in litter amber from other localities [88,89], leading to the inference that litter amber forms on the forested banks of estuarine rivers [30]. Although marine microfossils were not observed in the Otway amber, their absence does not exclude a similar depositional environment, but may indicate more distal deposition.

Opacity in the milky brown samples may be related to an abundance of microbubbles, e.g. [87,90], and/or pseudoinclusions, e.g. [30]. Similar opaque amber has been interpreted as root amber, which is typically large (20–30 cm^3^), lens-shaped, foliated, and exhibits a colour gradient from milky brown to transparent yellow, see [30,87]. A unique inclusion assemblage consisting of Type B pseudoinclusions, ectomycorrhizae and a limited number of plant inclusions in the absence of arthropods, pollen, spores and other microfossils may also be indicative of root resins, e.g. [28,30,87]. Although it is possible that the sample of milky brown amber collected from the Otway Basin was a fragment of a larger piece, either bisected by the coring process or eroded during transportation, the remaining diagnostic features of root resins could not be identified. As such, an inference regarding the depositional history of the milky brown amber is not made here.

## Conclusions

The Turonian amber collected from the Otway Basin represents the oldest amber occurrence from Australia and the southern-most occurrence from Gondwana. The amber contains bioinclusions including two plant spores (*Cyathidites minor* and *Kraeuselisporites* sp?), filamentous microorganisms and degraded organic matter, as well as a variety of pseudoinclusions. The presence of abundant particulate debris and wind-transported spores in the absence of aquatic organisms suggests that the Otway amber solidified subaerially. Orange, dark orange and red ambers are particularly rich in wind-transported debris and are generally inferred to have formed on tree trunks, whereas yellow amber is devoid of particulate debris and is inferred to have formed on upper tree branches. Based on the most common types of pseudoinclusions preserved in each amber colour, most yellow, red and dark orange ambers are inferred to represent immobile resins whereas most orange ambers are inferred to represent mobile resins. Finally, dark brown samples rendered opaque by an abundance of degraded organic matter are inferred to represent mobile resins that dripped off the tree and solidified on the ground surface. Although oxidation is known to darken the outer surface of amber and accounts for some of the colour variation observed in the Otway Basin, most colours are associated with specific assemblages of inclusions, which, in turn, is attributed to resin solidification at different levels on or adjacent to trees.
